# Influence of air temperature on the first flowering date of *Prunus yedoensis* Matsum

**DOI:** 10.1002/ece3.442

**Published:** 2014-01-03

**Authors:** Peijian Shi, Zhenghong Chen, Qingpei Yang, Marvin K Harris, Mei Xiao

**Affiliations:** 1 Key Laboratory of Sustainable Development of Marine Fisheries, Ministry of Agriculture, Yellow Sea Fisheries Research Institute, Chinese Academy of Fishery SciencesQingdao, 266071, China; 2 Wuhan Institute of Heavy Rain, China Meteorological AdministrationWuhan, 430074, China; 3 College of Forestry, Jiangxi Agricultural UniversityNanchang, 330045, China; 4 Department of Entomology, Texas A&M UniversityCollege Station, TX, 77843; 5 College of Resource and Environmental Sciences, Wuhan UniversityWuhan, 430072, China

**Keywords:** Accumulated degree days (ADD), base temperature, correlation coefficient, root mean square error, starting date

## Abstract

Climate change is expected to have a significant effect on the first flowering date (FFD) in plants flowering in early spring. *Prunus yedoensis* Matsum is a good model plant for analyzing this effect. In this study, we used a degree day model to analyze the effect of air temperatures on the FFDs of *P. yedoensis* at Wuhan University from a long-time series from 1951 to 2012. First, the starting date (=7 February) is determined according to the lowest correlation coefficient between the FFD and the daily average accumulated degree days (ADD). Second, the base temperature (=−1.2°C) is determined according to the lowest root mean square error (RMSE) between the observed and predicted FFDs based on the mean of 62-year ADDs. Finally, based on this combination of starting date and base temperature, the daily average ADD of every year was calculated. Performing a linear fit of the daily average ADD to year, we find that there is an increasing trend that indicates climate warming from a biological climatic indicator. In addition, we find that the minimum annual temperature also has a significant effect on the FFD of *P. yedoensis* using the generalized additive model. This study provides a method for analyzing the climate change on the FFD in plants' flowering in early spring.

## Introduction

Temperature can significantly affect developmental rate in poikilothermic animals and plants (Sharpe and DeMichele 1977; Schoolfield et al. 1981; Craufurd et al. 1996; Ikemoto 2005; Trudgill et al. 2005). In the mid-temperature range, the relationship between developmental rate and temperature approximates linearity (Campbell et al. 1974; Shi et al. 2011). The linear model is widely used to describe the temperature-dependent developmental rates:


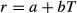
(1)

Here, *r* represents development rate (=1/*D*, where *D* represents developmental time required for completing a specific developmental stage) at constant temperature *T*; *a* and *b* are constants. There are two important thermal parameters related to the aforementioned linear model: the lower developmental threshold (LDT = *−a/b*) (also referred to as the base temperature, *T*_base_, in the degree day model) and the sum of effective temperatures (SET =* *1*/b*). The LDT represents the temperature at which developmental rate equals zero, that is, the intersection between the straight line of developmental rate and *x*-axis; the SET represents the accumulated degree days (ADD) required for completing a specific developmental stage. For different developmental stages of the same species, the SETs might be different, but the LDTs were demonstrated to be identical for most insects and mites (Jarošík et al. 2002, 2004; Kuang et al. 2012). Eq. 1 can be rewritten as follows:


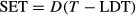
(2)

Here, *T* is constant temperature. When we attempt to use variable air temperature (as a function of time) to calculate ADD, the following equation is recommended (Aono 1993; Marletto et al. 1992; Lopez and Runkle 2004):


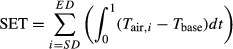
(3)

Here, *T*_air, *i*_ represents variable air temperature on the *i*-th day, which is a function of time *t* within 1 day; SD represents the starting date for a specific biological event; ED represents the ending date for this specific biological event; *T*_base_ is, as mentioned above, an equivalent concept of LDT. The integral symbol can be dropped if the fixed daily average temperature is used. For some insects and mites with small body size and short life cycle, the LDT and SET can be directly estimated by observing developmental times at different constant temperatures in the lab based on Eq. 1. However, many plants are larger and have a longer life cycle. Thus, these two thermal parameters, in general, cannot be obtained from an experiment of temperature-dependent development. Therefore, Eq. 3 is widely used to estimate these two thermal parameters without a requirement for setting different constant-temperature environments. In practice, a starting date and a base temperature of development are needed to be known to calculate the ADD required for reaching a specific biological event of plants.

The influence of air temperature from weather data on the first flowering date (FFD) in plants, especially the woody *Prunus* trees, has received much attention (Lindsey and Newman 1956; Lindsey 1963; Aono 1993; Fitter et al. 1995; Wielgolaski 1999; Ho et al. 2006; Chen et al. 2008; Miller-Rushing et al. 2008). Japanese cherry blossom (*Prunus yedoensis* Matsum) is an important ornamental plant in East Asia. Some studies have been carried out to explore the effect of air temperature in winter and early spring on the FFD of this plant (Aono 1993; Ho et al. 2006; Chen et al. 2008; Ohashi et al. 2012). Aono (1993) reported that the base temperature of *P. yedoensis* in Japan ranged from −2 to 6°C using the method of lowest root mean square errors (RMSEs) in days between the observed and predicted FFDs based on the mean of ADDs for some standard years; Ho et al. (2006) drew a conclusion that the base temperature of *P. yedoensis* in Seoul, Korea was 5.8°C by minimizing the standard deviation of ADDs; Ohashi et al. (2012) directly provided an empirical estimate of 5°C as the base temperature of *P*. *yedoensis*.

We provide a new method for estimating the starting date and base temperature of *P. yedoensis* based on the extensive meticulous FFD observations by Prof. Yihua Xiao and his daughter, Prof. Mei Xiao, in Wuhan University (30°32′21″N, 114°21′42″E) from 1951 to 2012. On the basis of the estimates of starting date and base temperature, we explored the influence of air temperature on the FFDs of *P. yedoensis*.

## Materials and methods

### FFD data of *P. yedoensis*

*Prunus yedoensis* in Wuhan University can date back to the Second World War. The Japanese army occupied Wuhan City and planted 28 *P. yedoensis* in Wuhan University in 1939. Additional plantings were made in 1957, 1985, and since the 1990s. Prof. Yihua Xiao began to record the FFD of *P. yedoensis* in 1947. And the observations have been continuously maintained since. After Prof. Yihua Xiao died, his daughter, Prof. Mei Xiao continued this study, making daily observations from March to April each year. The FFDs were ascertained when the number of full-opening flowers on a tree ≥3. The FFD data from 1947 to 1950 were not used because the weather data during these years are unavailable.

### Weather data

The daily minimum, maximum, and average air temperatures in Wuhan City (30°37′24′′N, 114°08′40′′E) from 1951 to 2012 were collected from the website of the China Meteorological Data Sharing Service System (http://cdc.cma.gov.cn/).

### Degree day model

We sought to find a starting date, a base temperature, and the ADD required for reaching the FFD. First, we set a wide combination of starting dates (from 1 January to the earliest FFD during 62 years) and base temperatures (from −6 to 6°C). Second, we calculated the ADDs for all combinations of starting date and base temperature by the proposed method listed in UC IPM Online (http://www.ipm.ucdavis.edu/WEATHER/ddconcepts.html). This generated a comprehensive set of candidate degree day models for further inspection.

Air temperature (*T*_air_) was defined as a function of time *t* (Shi et al. 2012):


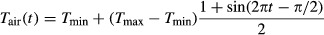
(4)

Here, *T*_min_ represents the daily minimum air temperature; *T*_max_ represents the daily maximum air temperature. On the latest FFD during 62 years, the daily maximum air temperature is only 25.3°C, which is not too high to affect the development of *P. yedoensis*. In general, there is an upper developmental threshold to terminate development in poikilothermic animals and plants. This upper developmental threshold is usually around 30°C. Thus, we did not consider the upper developmental threshold of *P. yedoensis* flowering in early spring. However, we need to consider the LDT (i.e., base temperature). When *T*_air_ ≥* T*_base_, the effective temperatures were accumulated.

The candidate models were inspected by obtaining the correlation coefficient derived from comparing the daily average ADD over the days from the starting date to the FFD for each subset of models that shared a given base temperature. The starting date corresponding to the lowest negative correlation coefficient was chosen as the best estimate of starting date for each of the 13 model sets representing all base temperatures. The most frequent starting date found among the 13 data sets was chosen as the final estimate of starting date.

Using the starting date estimated by the previous step, we analyzed which of the remaining candidate models had the lowest RMSE in days between the observed and predicted 62-year FFDs. For any combination of starting date and base temperature, the 62-year ADDs from the starting date and the FFD could be calculated. Then, we used the mean of 62-year ADDs as the critical value of ADDs to calculate the predicted FFD each year (Ring and Harris 1983; Aono 1993). For checking whether the final estimates of starting date and base temperature are accurate, we also inspected the isoline plot of RMSE in days to see the distribution of RMSE for different combinations of starting date and base temperature.

Finally, as the starting date and base temperature have been estimated by the above steps, the daily average ADD over the days from the starting date to the FFD each year was then ascertained. We tested whether there is a linear relationship between the daily average ADD and year. If there is a significant warming trend in climate, we hypothesized that the slope item should be statistically significant. The daily average ADD, as a biological climatic indicator, is different from that of the daily average temperature (over the days from the starting date to the FFD). The former has a more explicit biological relevance than the latter because the latter is based on the empirical meteorological record only. The former is linked with the ADD for *P. yedoensis* to develop.

### Considering the effect of minimum annual temperature on the FFD

Considering that the minimum annual temperature might affect the FFD of *P. yedoensis*, we used the generalized additive model (Hastie and Tibshirani 1990; Chambers and Hastie 1991) with two predictors to describe the FFD. These two predictors are as follows: 1) *x*_1_, the daily average ADD over the days from the starting date to the FFD; and 2) *x*_2_, the minimum annual temperature.


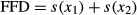
(5)

Here, *s*(·) is the smoothing function. As these effects of predictors might be linear, the multiple linear regression was also used to compare the effects with those from the generalized additive model.


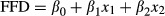
(6)

R version 2.15.0 (2012-03-30) (http://www.r-project.org/) was used to perform all the calculations. And the package of “mgcv” was used to fit the generalized additive model.

## Results

### Starting date and base temperature in the degree day model

**Figure 1:**
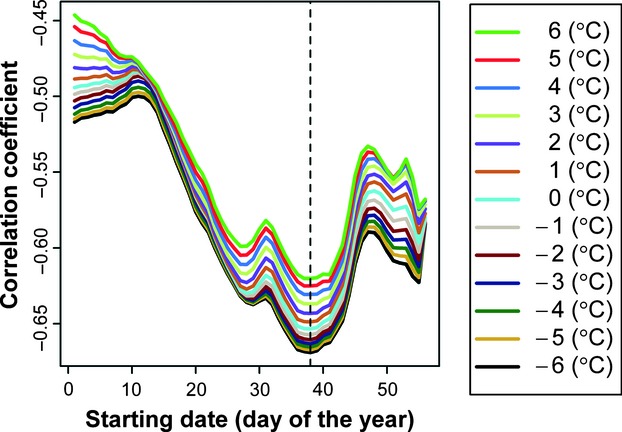
Correlation coefficients between the FFDs and the daily average ADDs for different combinations of starting date and base temperature. The vertical dashed line represents the starting date of day 38.

Figure 1 displays the correlation coefficients between the daily average ADD and the FFD for different combinations of starting date and base temperature. When the starting date = day 38 (day of the year), the correlation coefficient reaches its minimum at each given base temperature. When the base temperatures are ≤0°C, there are only small differences among the minimum correlation coefficients. That is, results indicate 7 February (i.e., day 38) for the starting date, and show the base temperature might be no more than 0°C.

**Figure 2:**
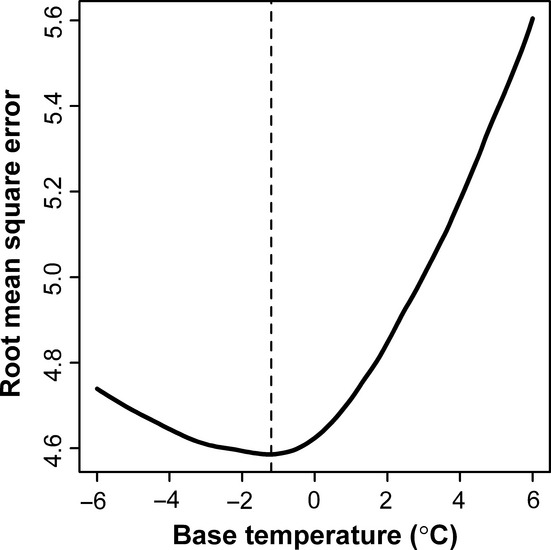
RMSEs for the combinations of base temperatures (ranging from −6 to 6°C in 0.1°C increments) and a fixed starting date of day 38. The vertical dashed line represents the base temperature of −1.2°C.

Fixing the starting date at day 38, we calculated the RMSEs in days for the different base temperatures (Fig. 2). When the base temperature = −1.2°C, the corresponding RMSE is the lowest (=4.586). Thus, the best estimate of base temperature is set at −1.2°C.

**Figure 3:**
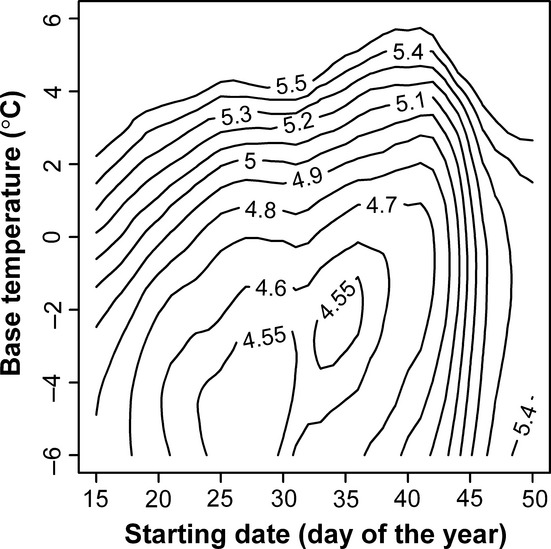
The RMSE isolines for different combinations of starting date and base temperature.

We also calculated all RMSEs in days for different combinations of starting date and base temperature (Fig. 3). Smaller than 4.6 RMSE isoline, there are two peaks of RMSE isolines (<4.6 but >4.5). However, ideal RMSE isolines should have only one peak. Thus, these two peaks should not be used to accurately estimate the starting date and base temperature. The combination of starting date of day 38 and base temperature of −1.2°C can result in a low RMSE of 4.586 (also <4.6). In fact, the difference in RMSEs between any combination in either peaks and the current combination of a starting date of day 38 and a base temperature of −1.2°C is very small, because the lowest RMSE from all combinations is still higher than 4.5. The correlation coefficient corresponding to the starting date of day 38 is −0.658 (*P *<* *0.05).

**Figure 4:**
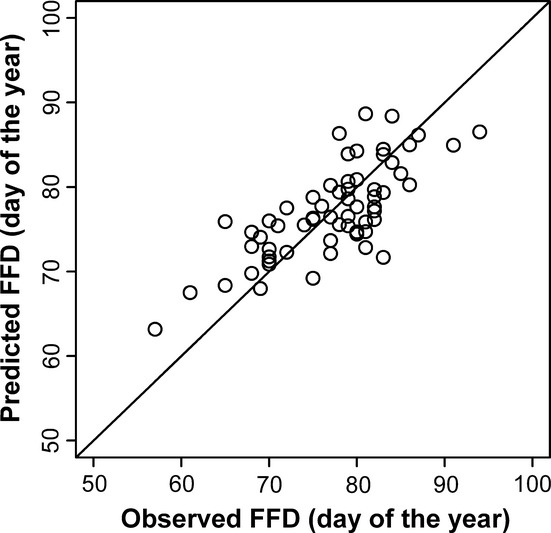
The comparison between the observed and predicted FFDs by the degree day model.

Figure 4 shows the comparison between the observed and predicted FFDs using the degree day model. The correlation coefficient between them is 0.7511.

### Effect of the minimum annual temperature on the FFD

**Figure 5:**
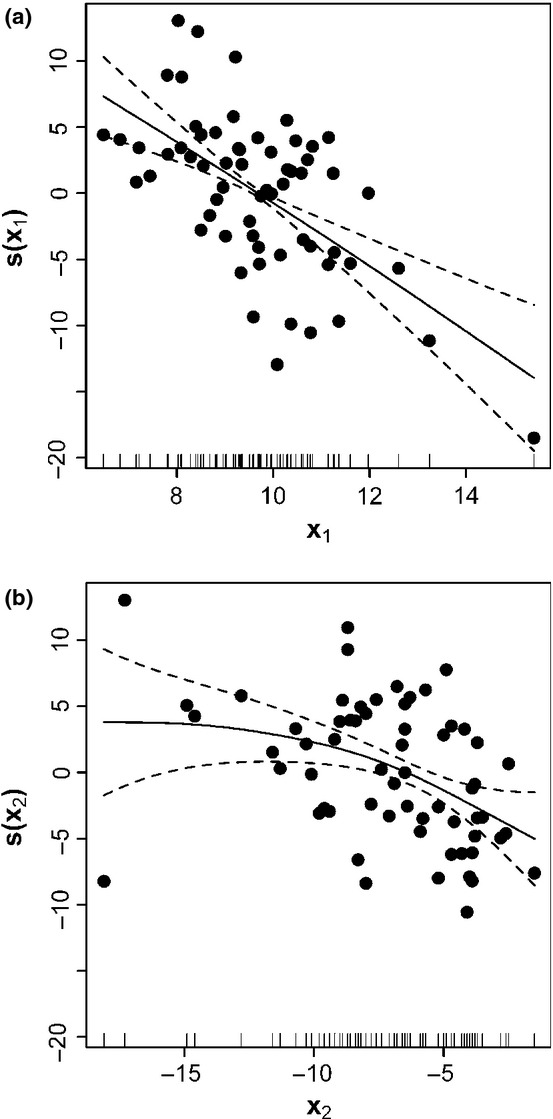
Generalized additive fit of the FFD to two predictors: (a) *x*_1_, the daily average ADD (over the days from the starting date to the FFD); and (b) *x*_2_, the minimum annual temperature. The dashed curves are pointwise twice standard-error bands. Each panel represents the contribution of that predictor to the fitted value. The points represent the partial residuals.

Using the generalized additive model, the two predictors are statistically significant (*P *<* *0.05 for both). The adjusted coefficient of determination (*R*_adj_^2^) = 0.513, and deviance explained = 53.7%. Figure 5 displayed the fit. The daily average ADD over the days from the starting date to the FFD has an obvious linear effect on the FFD; the minimum annual temperature has a non-linear effect on the FFD. However, the latter effect can approximate linearity. We also used multiple linear regression to analyze the effects of these two predictors on the FFD (Table 1). *R*_adj_^2^ = 0.4898; *F*_(2, 59)_ = 30.28; *P *=* *8.96 × 10^−10^ < 0.05. The multiple linear model also can describe the effects of the daily average ADD and the minimum annual temperature on the FFD very well. These two predictors both have a negative effect on the FFD.

**Table 1:** Multiple linear fit of the FFD to two predictors

	Estimate	Standard error	*t* value	Pr(>*t*)
*β*_0_	97.0151	4.9379	19.647	<2 × 10^−16^^*^^*^^*^
*β*_1_	−2.4834	0.4367	−5.687	4.25 × 10^−7^^*^^*^^*^
*β*_2_	−0.5792	0.1956	−2.960	4.42 × 10^−3^^*^^*^

### Is there evidence of climate warming?

**Figure 6:**
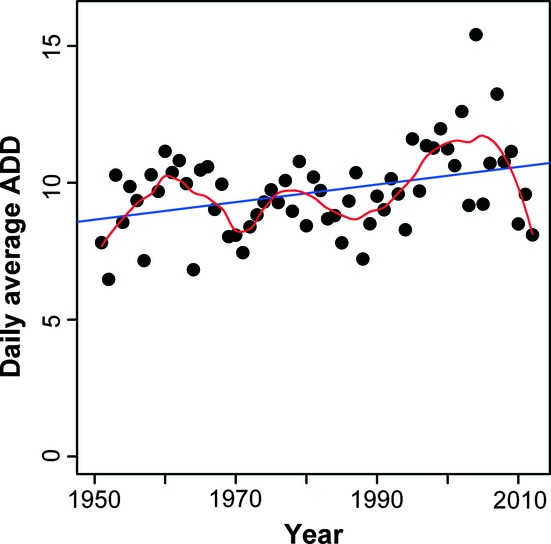
Linear fit of the daily average ADD to year. Any a daily average ADD was calculated based on the combination of a starting date of day 38 and a base temperature of −1.8°C. The points represent 62-year daily average ADDs; the straight line is obtained by the linear regression; and the curve is obtained by the local regression (loess).

Carrying out a linear fit of the daily average ADD (over the days from the starting date to the FFD) to year, the linear model's slope is statistically significant (*P *=* *0.0031 < 0.05). *R*_adj_^2^ = 0.1223. There is an increasing trend for the daily average ADD, as a biological climatic indicator (see Table 2 and Fig. 6).

**Table 2:** Linear fit of the daily average ADD to year

	Estimate	Standard error	*t* value	Pr(>*t*)
(Intercept)	−53.9092	20.6294	−2.613	0.0113^*^
Year	0.0321	0.0104	3.082	0.0031^*^^*^

## Discussion

### Comparing other methods for estimating the starting date and base temperature

**Figure 7:**
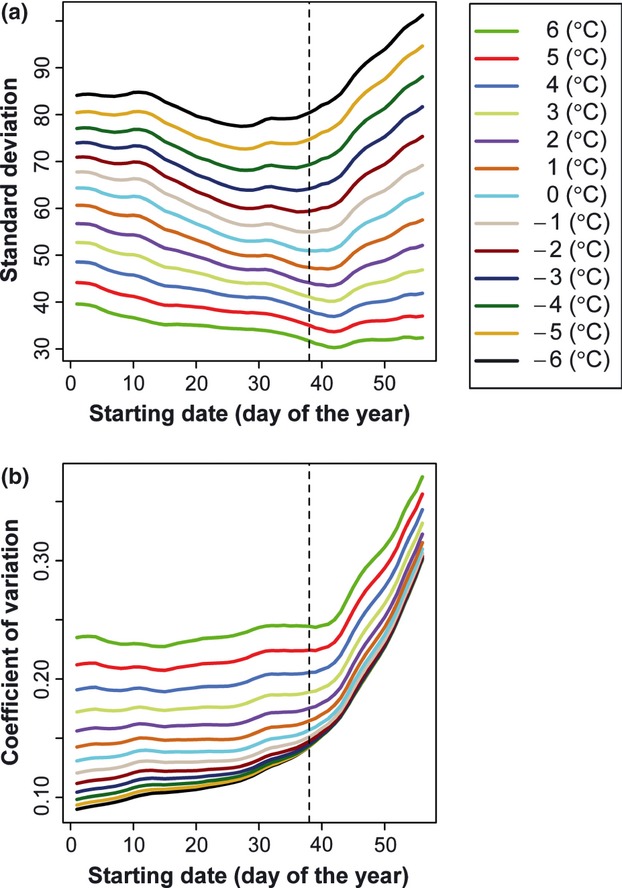
Standard deviation and coefficient of variation in ADDs. (a) Standard deviations for the base temperatures range from −6 to 6°C in 1°C increment; (b) Coefficients of variation for the base temperatures range from −6 to 6°C in 1°C increment. The vertical dashed line represents the starting date of day 38.

Previous investigators (e.g., Lindsey and Newman 1956; Lindsey 1963; Boyer 1973) suggested using the coefficient of variation of ADDs or the standard deviation of ADDs to determine the starting date and base temperature. Ho et al. (2006) also suggested using the standard deviation of ADDs to attain this objective. However, in our opinion, it is very difficult to use these two methods to accurately determine the starting date and base temperature. Figure 7 illustrates the standard deviation and coefficient of variation in ADDs from the current data. From this figure, we cannot clearly estimate the starting date. Although the standard deviations for some base temperatures can reach their lowest values around day 40 (Fig. 7(a)), the starting dates are predicted to be different for different base temperatures. And there are break points (beyond which there is a rapid increase for the curve of coefficient of variation vs. starting date) when the starting day approximates day 40 from Fig. 7(b), but we also cannot clearly ascertain the accurate starting date. Relative to these two methods, the lowest correlation coefficient method (see Fig. 1) can clearly determine the starting date. During the range having biological meaning for *P. yedoensis* from −6 to 6°C, all the lowest correlation coefficients occur when the starting date = day 38 (namely 7 February each year). Ho et al. (2006) obtained a combination of starting date of day 36 and base temperature of 5.8°C for *P. yedoensis* in Seoul, Korea based on the method of the lowest standard deviation of ADDs. According to Fig. 7(a), when the base temperature was set higher than −1.2, the standard deviation would be smaller. That is, the base temperature would be found at the highest preliminary value on the condition that the ADD did not equal 0. Thus, to use the method of standard deviation or that of coefficient of variation to estimate the starting date and base temperature is not feasible. At least, they failed in accurately estimating the starting date and base temperature. The final estimates will result in a larger RMSE between the observed and predicted FFDs.

### Some factors presumedly affecting the predicted results

Some investigators (Ring and Harris 1983; Aono 1993) obtained a lower RMSE in days using the degree day model. Ring and Harris (1983) calculated the starting date of day 72 and base temperature of 3.3°C for the 50% emergence of the female pecan nut casebearer (*Acrobasis nuxvorella* Nuenzig) using the data of 1918–1923. The corresponding RMSE equals 3.42. They only used 6-year data, which is presumedly the reason of obtaining a small RMSE. Aono (1993) obtained a group of RMSEs in days ranging from 1.37 to 3.06 for the FFDs of *P. yedoensis* in different sites of Japan. The recorded years are 1961–1985, that is, 25 years. In this study, the recorded years in Wuhan University are 1951–2012, that is, 62 years. It is natural that the RMSE of 4.586 calculated in this study is larger than the report from Aono (1993) because the longer recorded time means more climatic variation, especially the daily maximum and minimum air temperatures in winter and early spring among years, which further leads to larger annual differences in the FFDs. In fact, Ho et al. (2006) used a 83-year FFD data set of *P. * *yedoensis*, and the RMSE based on the combination of starting date of day 32 and base temperature of 5.8°C is also shown to be large (see Fig. 4(c) published in Ho et al. 2006).

Miller-Rushing et al. (2008) stated that population size and sampling frequency both had significant effects of the FFD in plants. An increased density might show an earlier FFD than that of a smaller population (see Fig. 1 published in Miller-Rushing et al. 2008 for further details). However, in Wuhan University, the population size of *P. yedoensis* is relatively stable as this plant could not naturally increase its population size. And sampling frequency also has little effect on the FFD in this site because the observation method is consistent during the past 62 years.

There is another important factor that might substantially affect the model prediction. That is the distance of 22.8 km between Wuhan Weather Bureau (where the climate site is located) and Wuhan University. The urban heat island intensity in Wuhan City has been demonstrated to exhibit an asymmetrical change (Chen et al. 2007). Excluding the effect of the urban heat island, 22.8 km may have some effects on daily air temperature difference between Wuhan Weather Bureau and Wuhan University. Even so, the daily air temperature data of Wuhan Weather Bureau still, in general, reflect that in Wuhan University. Using the degree day model or using the generalized additive model has shown a satisfactory goodness-of-fit (*R*^2^ > 0.50). However, if the climate site was nearer to Wuhan University, it might further improve the goodness-of-fit.

### Lower developmental threshold of *P. yedoensis*

In general, we refer to –*a/b* based on Eq. 1 as the lower developmental threshold (LDT), but as noted above, it is the equivalent concept of base temperature (*T*_base_) in the degree day model. Interestingly, Lindsey and Newman (1956) stated that the average meteorological threshold determined (i.e., base temperature) was slightly higher than thresholds determined by physiologists in constant-temperature experiments (i.e., LDT). To our experience, the LDTs of insects and mites usually range from 5 to 15°C (also see Kiritani 2012). Leach et al. (1986) reported that the LDT of the rhabditid nematode (*Goodeyus ulmi* Goodey) is 1.3°C. Craufurd et al. (1996) reported that the LDTs for 50% seed germination of 12 cowpea genotypes (*Vigna unguiculata* L.) ranged from 6 to 12°C. However, there is no direct experimental evidence to prove that the LDTs for the FFDs of plants' flowering in early spring also range from 5 to 15°C. In this study, the developmental time up to the FFD does not only denote the developmental process of flowering. It should include the time from breaking dormancy in winter to the FFD, so the ADD required should be large. Based on the combination of a starting date of day 38 and a base temperature of −1.2°C, the daily average ADD for 62 years equals 371.5. We believe that the LDT of *P. yedoensis* is relatively low (below 0°C). This conclusion is different from the studies of Ho et al. (2006) and Ohashi et al. (2012). However, it is located in the range from −2 to 6°C reported by Aono (1993). We must point out that the LDT for a plant is actually not a real physiological temperature. It means the lowest environmental temperature at which a plant can bear for development. For different plants, their body sizes are different that might lead to the difference for bearing coldness. In fact, the concept of LDT is an ecological terms. We should not equate it to a physiological temperature, because the water will become ice below zero degree. However, for a plant, when the air temperature is below zero degree, the inner temperature in a plant should be high than zero degree.

Previous studies have neglected the correlation coefficients between the daily average ADD and the FFD for different combinations of starting date and base temperature. Some studies have used the correlation coefficients between the daily (or monthly) average temperature and the FFD for different combinations of starting date and base temperature. However, this study implies that a better choice is to use the daily average ADD. We determined the starting date by observing the minimal correlation coefficient. Then, we suggested determining the base temperature using the least RMSE method based on the given starting date. These two steps can predict the FFDs of *P. yedoensis* very well. However, whether this method can be applied to other species deserves further investigation.
